# B Cells Play Key Roles in Th2-Type Airway Immune Responses in Mice Exposed to Natural Airborne Allergens

**DOI:** 10.1371/journal.pone.0121660

**Published:** 2015-03-24

**Authors:** Li Yin Drake, Koji Iijima, Kenichiro Hara, Takao Kobayashi, Gail M. Kephart, Hirohito Kita

**Affiliations:** 1 Department of Internal Medicine, Division of Allergic Diseases, Mayo Clinic, Rochester, Minnesota, United States of America; 2 Department of Immunology, Mayo Clinic, Rochester, Minnesota, United States of America; Virginia Tech University, UNITED STATES

## Abstract

Humans are frequently exposed to various airborne allergens. In addition to producing antibodies, B cells participate in immune responses via various mechanisms. The roles of B cells in allergic airway inflammation and asthma have been controversial. We examined the functional importance of B cells in a mouse model of asthma, in which mice were exposed repeatedly to common airborne allergens. Naïve wild-type BALB/c mice or B cell-deficient JH^−/−^ mice were exposed intranasally to a cocktail of allergen extracts, including *Alternaria*, *Aspergillus*, and house dust mite, every other day for two weeks. Ovalbumin was included in the cocktail to monitor the T cell immune response. Airway inflammation, lung pathology, and airway reactivity were analyzed. The airway exposure of naïve wild type mice to airborne allergens induced robust eosinophilic airway inflammation, increased the levels of Th2 cytokines and chemokines in the lung, and increased the reactivity to inhaled methacholine. These pathological changes and immune responses were attenuated in B cell-deficient JH^−/−^ mice. The allergen-induced expansion of CD4^+^ T cells was impaired in the lungs and draining lymph nodes of JH^−/−^ mice. Furthermore, lymphocytes from JH^−/−^ mice failed to produce Th2 cytokines in response to ovalbumin re-stimulation *in vitro*. Our results suggest that B cells are required for the optimal development of Th2-type immune responses and airway inflammation when exposed to common airborne allergens. The therapeutic targeting of B cells may be beneficial to treat asthma in certain patients.

## Introduction

Asthma is a complex and heterogeneous disease of the airways that affects millions of people worldwide. Airway inflammation in asthma is often triggered by the exposure to environmental allergens, such as molds and arthropods. Concurrent exposure to several airborne allergens is common [1], and exposures to multiple allergens are significantly associated with asthma development [2]. Certain allergens, such as *Alternaria* and the house dust mite (HDM), are detected together in the home environment [2]. Animal models of asthma that reflect the natural environmental exposure in humans may provide valuable information to understand the pathophysiological mechanisms of the disease.

Th2 CD4^+^ T cells are thought to be the central immune cell that regulates allergic airway inflammation in asthma [3,4]. Other T-cell subsets and innate immune cells may also be involved [5]. On the other hand, the functional roles of B cells in allergic airway inflammation have been controversial, whereas their ability to produce the IgE antibody is well established. For example, in mouse models of asthma that use ovalbumin (OVA) as the antigen, B cells are required for airway hyperreactivity (AHR) but not for eosinophilic airway inflammation or the production of Th2 cytokines [6]. Minimal differences in airway inflammation are observed in wild-type (WT) mice and B cell-deficient mice that were sensitized and challenged with the fungus, *Aspergillus fumigatus* [7,8]. In contrast, the exposure of B cell-deficient mice to cockroach allergens decreases airway levels of Th2 cytokines but does not affect the number of eosinophils in the airway [9]. Therefore, the roles of B cells in allergic immune responses may vary depending on immunization protocols and the nature of the allergens.

To mimic natural allergen exposure in humans, we recently developed a mouse model in which animals are simultaneously exposed to several common airborne allergens for a prolonged period of time [10]. In this model, the chronic intranasal exposure of naïve animals to a cocktail of natural allergen extracts, including *Alternaria*, *Aspergillus*, and HDM, triggers robust Th2-type immune responses and eosinophilic airway inflammation, and the allergens work synergistically to induce a strong response [10]. Marked airway infiltration of both CD4^+^ cells and B cells are also observed in these mice, and adaptive immunity, presumably CD4^+^ T cells, is necessary for developing pathological changes. In the current study, we examined the roles of B cells by using a similar model. We found that exposure to natural allergens induced airway inflammation and lung pathology, and B cells played key roles in these responses. Importantly, the expansion and differentiation of allergen-specific CD4^+^ T cells were attenuated in B cell-deficient mice.

## Materials and Methods

### Mice and allergens

BALB/cJ and BALB/c-JH^-/-^ mice were obtained from the Jackson Laboratory (Bar Harbor, ME). Female mice (6–12 weeks old) were used in all experiments. All animal experiments and handling procedures were approved by the Mayo Clinic Institutional Animal Care and Use Committee and performed according to its guidelines. The allergen extracts, including *Alternaria*, *Aspergillus fumigatus*, and HDM, were purchased from Greer Laboratories (Lenoir, NC). Endotoxin was undetectable in all of these extracts (<10 ng/mg extract). Endotoxin-free OVA was prepared in our laboratory, as previously described [11].

### Airway allergen exposure model

Mice were lightly anesthetized with isoflurane and administered intranasally with a cocktail of allergen extracts in 50 μl of endotoxin-free phosphate-buffered saline (PBS), including 10 μg each (total weight) of *Alternaria*, *Aspergillus*, and HDM. To monitor the development of the T-cell immune response, the allergen cocktail was pulsed with 10 μg OVA. Control mice received 50 μl PBS alone. Mice were exposed to allergens on days 0, 2, 4, 7, 9, 11, and 14. Twenty-four hours after the last allergen exposure, the AHR to inhaled methacholine was assessed in conscious mice by using whole-body plethysmography (Buxco Electronics Ltd, Sharon, CT), as described previously [12]. Briefly, mice were exposed for 3 min to aerosolized PBS, followed by incremental doses of aerosolized methacholine, and AHR was represented as a percentage of baseline enhanced pause (Penh) values before PBS exposure. After AHR assessments, blood samples were collected from these mice. The mice were then euthanized by intraperitoneal injection of pentobarbital, and bronchoalveolar lavage (BAL) fluid and lungs were collected. Cell numbers in BAL fluids were counted, and cell differentials were determined in Wright-Giemsa-stained cytospin slides. A part of the lungs was homogenized for cytokine and chemokine analyses, and the reminder of the lungs was fixed in 10% formalin and embedded in paraffin for pathological analysis. The levels of cytokines (IL-4, IL-5, IL-13 and IFN-γ) and chemokines (C-C motif ligand [CCL]11 and CCL24) in the supernatants of BAL fluids and lung homogenates were analyzed by ELISA (R&D Systems, Minneapolis, MN), following the manufacturer’s instructions. Lung sections were stained with hematoxylin and eosin and the periodic acid-Schiff stain.

### Immunohistochemistry

To detect the eosinophil MBP in lung tissues, sections were deparaffinized and rehydrated. Endogenous peroxidase and alkaline phosphatase were blocked by treating the sections with a dual endogenous block (Dako, Carpinteria, CA) for 10 minutes. After washing with PBS, the sections were incubated with a pepsin solution (Life Technologies, Digest All 3, Grand Island, NY) for 10 minutes to unmask the antigenic sites. After another wash, sections were blocked with Background Sniper (BioCare Medical, Concord, CA) for 5 minutes. Sections were then washed and incubated overnight at 4°C with 4 μg/mL of either rat IgG1 (eBiosciences, San Diego, CA) or rat anti-mouse MBP (kindly provided by Dr. James J. Lee, Mayo Clinic Arizona, Clone MT-14.7), which was diluted in Da Vinci Green Diluent (BioCare Medical). Staining was visualized using a rat-on-mouse alkaline phosphatase-polymer detection kit (BioCare Medical) and a fast red chromogen kit (BioCare Medical), as per the manufacturer’s instructions. Sections were counterstained with methyl green (Vector Laboratories, Burlingame, CA) and mounted with Permount (Fisher, Waltham, MA).

### Analysis of lymphocytes in the lungs and draining lymph nodes

To obtain single-cell suspensions, lungs were minced and digested with a cocktail of collagenases (Roche Diagnostics, Indianapolis, IN) and DNase I (StemCell Technologies, Vancouver, BC, Canada) for 1 h at 37°C. Mediastinal lymph nodes (MLNs) were dispersed by using a cell strainer and syringe, and red blood cells were lysed with Ammonium-Chloride-Potassium lysing buffer. The cells were counted and then incubated with fluorescently labeled antibodies against mouse CD3 and CD4 (BD Biosciences, San Jose, CA). The stained cells were analyzed by a FACScan flow cytometer (BD Immunocytometry Systems, San Jose, CA), and the proportions of lymphocyte subsets were analyzed by gating electronically on lymphocytes. The gating strategies for lymphocytes in the lungs and MLNs are provided in Supporting Information S1 Fig.

Aliquots of single-cell suspensions of MLNs were suspended in RPMI 1640 medium (Invitrogen, Carlsbad, CA), which was supplemented with 10% fetal bovine serum, and cultured in 96-well round-bottom plates (5×10^5^ cells/ml) with or without OVA (100 μg/ml). Five days later, the culture supernatants were collected, and the cytokine levels were analyzed by ELISA, as described above.

### Statistical analysis

Data are presented as the mean±standard error of the mean (SEM), as indicated in the figure legends. Statistical significance was assessed with the Student’s *t* test, and p< 0.05 was considered significant.

## Results

### Allergen exposure-induced airway inflammation is diminished in B cell-deficient mice

**Fig 1:**
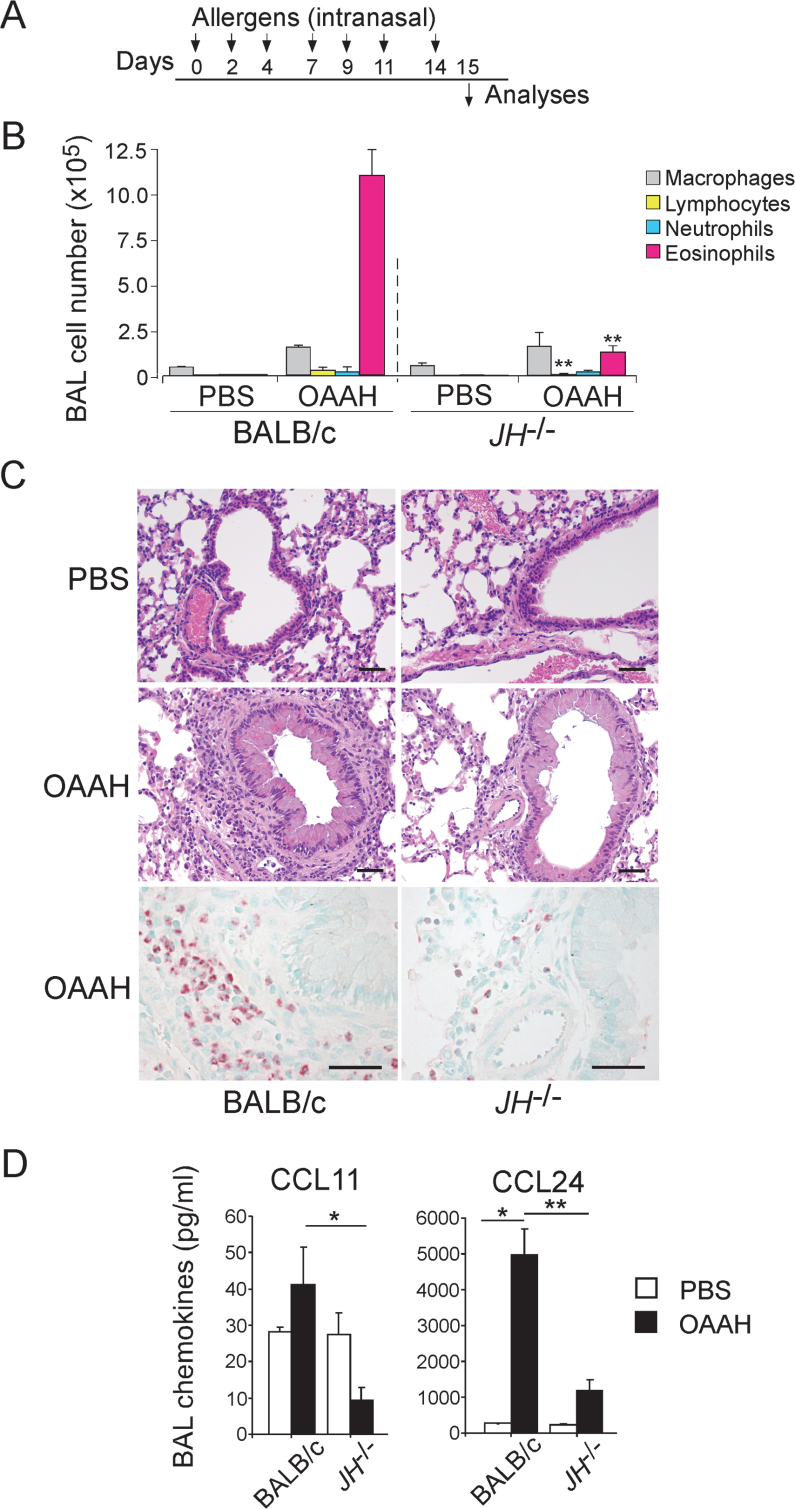
Allergen exposure-induced airway inflammation is attenuated in B cell-deficient JH^-/-^ mice. (A) The experimental protocol is shown. Wild-type (WT) or JH^-/-^ mice were intranasally exposed to a cocktail of allergens (OAAH; ovalbumin, *Alternaria*, *Aspergillus*, house dust mite) on days 0, 2, 4, 7, 9, 11, and 14, and they were analyzed on day 15. (B) Cell differentials in BAL fluids were counted and presented as mean±standard error of the mean (SEM; n = 4 and 6 in PBS and OAAH, respectively). **, p<0.01, between WT BALB/c and JH^-/-^ mice exposed to OAAH. (C) Lung sections were stained with hematoxylin and eosin stains (upper and middle panels) or with the anti-mouse MBP antibody (lower panels). Original magnification, 160×. Scale bars, 50 μm. Data are representative of two independent experiments. (D) Chemokine levels in BAL fluids were determined by ELISA. Results are presented as mean±SEM (n = 4 and 6 in PBS and OAAH, respectively).

To mimic allergen exposure in humans, we intranasally administered a combination of allergen extracts to naïve BALB/c mice 3 times a week for 2 weeks [10] (Fig. 1A). No systemic immunization, such as the intraperitoneal or subcutaneous injection of allergens, was performed throughout the procedure. For allergens, we used HDM, *Alternaria*, and *Aspergillus*, which are associated with treatment-resistant asthma and are commonly found in homes [2,13,14,15]. The cocktail of HDM, *Alternaria*, and *Aspergillus* extracts (10 μg/dose each) was spiked with endotoxin-free OVA (10 μg/dose), which allowed us to monitor the development of antigen-specific adaptive immunity. Therefore, the allergen mix was named OAAH (short for OVA, *Alternaria*, *Aspergillus*, and HDM).

Multiple exposures to OAAH induced a robust increase in BAL eosinophils at 2 weeks, and eosinophils made up approximately 80% of total BAL cells (Fig. 1B). OAAH exposure also induced significant increases in macrophages, lymphocytes, and neutrophils in BAL fluids. To determine whether B cells are required for this allergen-induced airway inflammation, we exposed B cell-deficient JH^-/-^ mice to OAAH. OAAH-treated JH^-/-^ mice showed significant decreases in the numbers of eosinophils and lymphocytes in BAL fluids, as compared with WT mice (Fig. 1B, p<0.01). No differences in macrophage and neutrophil numbers were observed.

Hematoxylin and eosin staining revealed that the lung of PBS-treated JH^-/-^ mice has normal histology, similar to that seen in PBS-treated WT mice (Fig. 1C, upper panels). OAAH exposure induced the peribronchial and perivascular infiltration of leukocytes in the WT mouse lung (Fig. 1C, middle panels). Thickening of the respiratory epithelium was also apparent with OAAH exposure. In contrast, the infiltration of inflammatory cells that were induced by OAAH exposure was decreased in JH^-/-^ mice, as compared to WT mice. To specifically analyze eosinophil infiltration, we stained lung sections with the anti-MBP antibody. In WT mice, OAAH exposure induced a significant increase in MBP-positive cells in peribronchial tissues (Fig. 1C, lower panels). The MBP-positive cells were apparently reduced in the lungs of JH^-/-^ mice. Collectively, these findings suggest that the repeated airway exposure of naïve mice to common airborne allergens induces airway eosinophilia. Importantly, this response was attenuated in B cell-deficient mice.

Chemokines, such as CCL11 and CCL24 (also known as eotaxin-1 and eotaxin-2, respectively), play important roles in the recruitment of eosinophils to inflamed tissues [16]. To examine the mechanism responsible for reduced allergen-induced airway eosinophilia in JH^-/-^ mice, we analyzed the chemokine levels. In WT mice, OAAH exposure induced a significant increase in the levels of CCL24 in BAL fluids (Fig. 1D, p<0.05). No significant changes in the levels of CCL11, which is likely expressed constitutively [16], were observed. The BAL fluid levels of both CCL11 and CCL24 were significantly lower in OAAH-treated JH^-/-^ mice than in OAAH-treated WT mice (p<0.05 and p<0.01 for CCL11 and CCL24, respectively). These data suggest that impaired chemokine production might be responsible for attenuated OAAH-induced airway eosinophilia in B cell-deficient mice.

### Development of AHR is reduced in B cell-deficient mice

**Fig 2:**
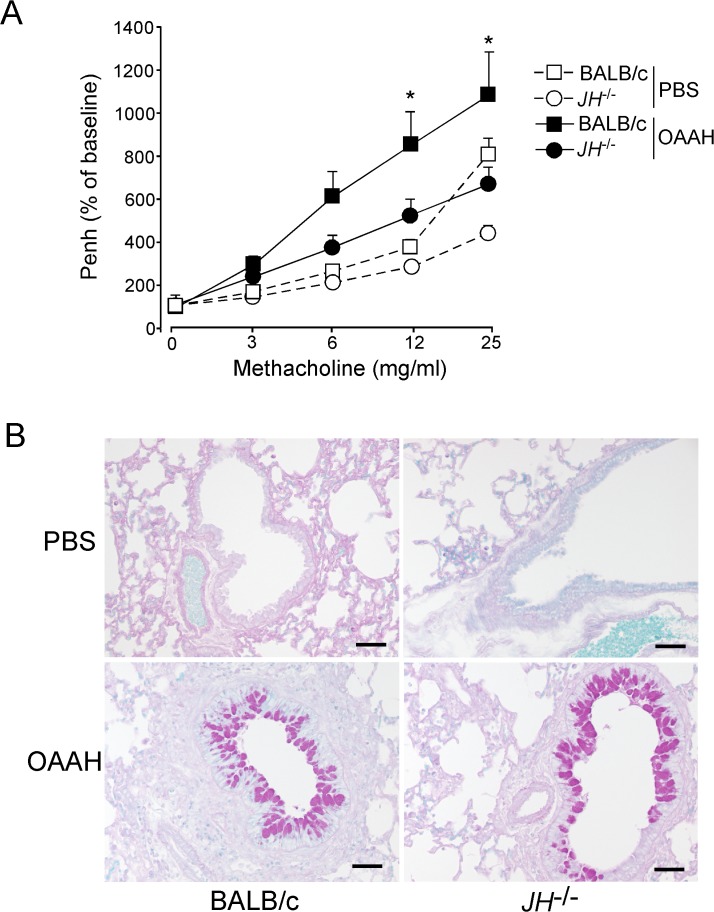
Airway reactivity is reduced in JH^-/-^ mice. WT or JH^-/-^ mice were intranasally exposed to OAAH or PBS for 2 weeks, as described in Fig. 1A. (A) Airway reactivity to inhaled methacholine was assessed by whole-body plethysmography, as described in the Methods section. Results are presented as the percentage of baseline (i.e. before PBS or methacholine challenge) and as mean±SEM (n = 4 and 6 in PBS and OAAH, respectively). *, p<0.05, between WT mice and JH^-/-^ mice exposed to OAAH. (B) Lung sections were stained with the periodic acid-Schiff stain. Original magnification, 160×. Scale bars, 50 μm. Data are representative of two independent experiments.

In mouse models of asthma, animals often display AHR to inhaled methacholine and mucus hyperplasia. To investigate the role of B cells in allergen-induced AHR, WT and JH^-/-^ mice were repeatedly exposed to OAAH, and the airway reactivity to methacholine was examined after 24 h. WT mice that were exposed to OAAH demonstrated increased methacholine responsiveness, as compared with PBS-treated WT mice (Fig. 2A). Methacholine responsiveness was also increased in OAAH-treated JH^-/-^ mice, as compared with PBS-treated JH^-/-^ mice (Fig. 2A). However, the magnitude of the increase in AHR in these mice was smaller, and the level of methacholine responsiveness in OAAH-treated JH^-/-^ mice was significantly lower than that in OAAH-treated WT mice (p<0.05 at 12 mg/ml and 25 mg/ml). When lung tissues were examined by periodic acid-Schiff staining, WT mice that were exposed to OAAH showed an increase in mucus production (Fig. 2B). However, no apparent differences were observed between OAAH-treated WT and JH^-/-^ mice.

### T-cell infiltration and cytokine production in the lungs are attenuated in B cell-deficient mice

**Fig 3:**
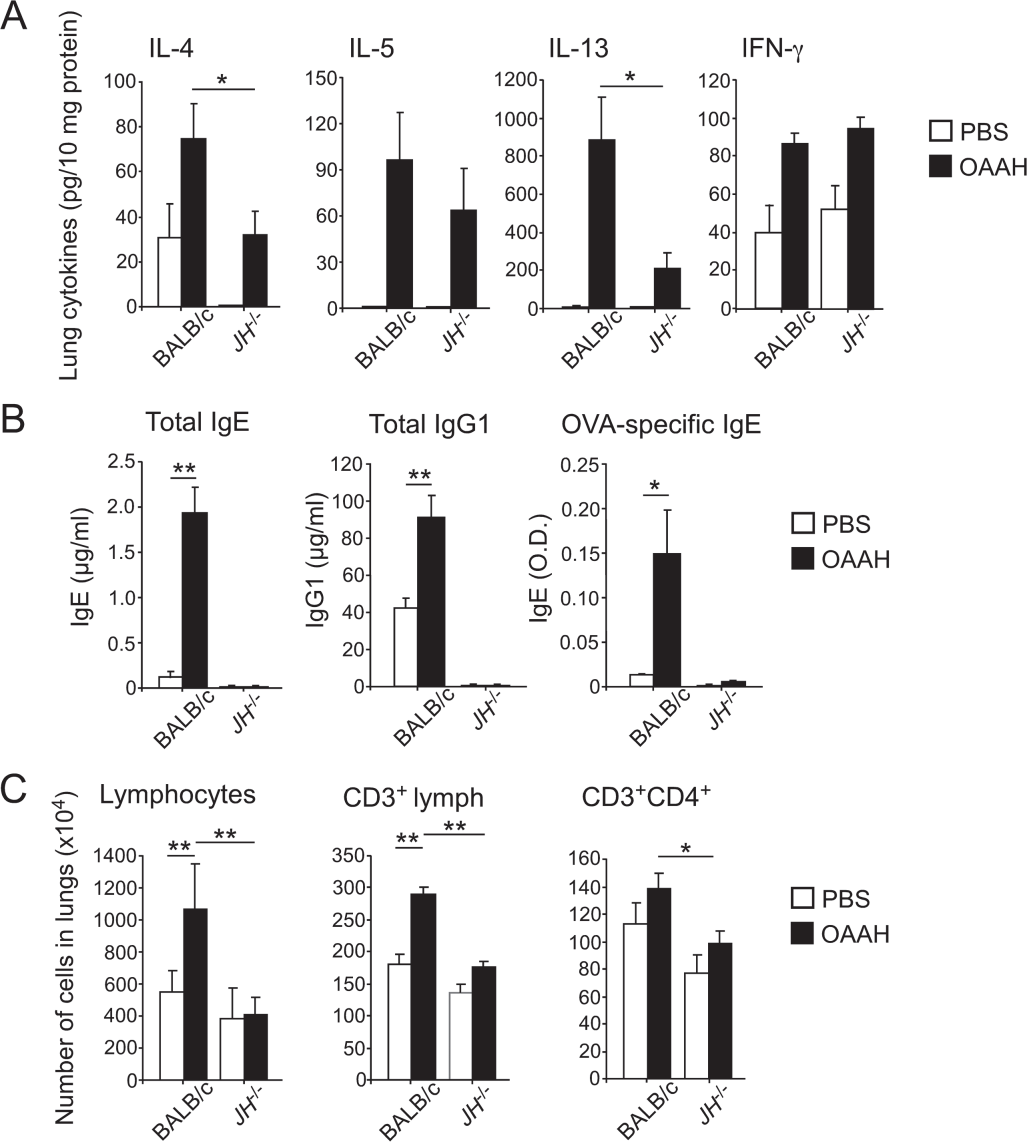
Th2-type cellular and humoral immune responses are decreased in JH^-/-^ mice. WT or JH^-/-^ mice were intranasally exposed to OAAH or PBS for 2 weeks, as described in Fig. 1A. (A) Cytokine levels in lung homogenates were determined by ELISA. (B) Blood was collected from these mice 24 hours after the last allergen exposure. The antibody levels in the plasma were analyzed by ELISA. (C) Single-cell suspensions of the lung were stained with anti-CD3 and anti-CD4 antibodies and analyzed by flow cytometry. Cell numbers were calculated based on cell counts and percentages of CD3^+^ and CD4^+^ cells. Results are presented as mean±SEM (n = 5 mice per group). *, p<0.05; **, p<0.01, between the groups indicated by horizontal lines. Data are representative of two independent experiments.

Because Th2 cells and their products, including Th2-type cytokines and chemokines, likely play key roles to regulate eosinophilic airway inflammation and pathology in asthma, we next examined the levels of Th2 cytokines in lung homogenates of PBS- or OAAH-treated mice. In WT mice, exposure to OAAH increased the levels of Th2 cytokines, including IL-4, IL-5, and IL-13, in the lungs (Fig. 3A). In B cell-deficient JH^-/-^ mice, OAAH exposure induced a modest increase in IL-4 and IL-13 levels, as compared with PBS-treated JH^-/-^ mice. The levels of these cytokines were significantly lower in OAAH-treated JH^-/-^ mice than in OAAH-treated WT mice (p<0.05). The levels of IL-5 were less affected than those of IL-4 or IL-13. The levels of a Th1-type cytokine, IFN-γ, were similar between WT and JH^-/-^ mice.

To examine the effects of allergen exposure on the levels of antibodies, we analyzed total and specific antibodies in plasma specimens. The levels of total IgE and total IgG1 significantly increased in WT mice exposed to OAAH allergens as compared to WT mice exposed to PBS (Fig. 3B, p<0.01). Furthermore, OVA-specific IgE antibody was detected in the plasma of OAAH-exposed WT mice. In contrast, no IgE or IgG1 antibodies were detectable in OAAH-exposed JH^-/-^ mice.

These observations led us to suspect that B cells or antibodies are necessary for the expansion and/or recruitment of CD4^+^ T cells to the lungs. To address this, we analyzed lymphocyte populations in the lung tissues by FACS. The lung of PBS-treated JH^-/-^ mice contained roughly comparable numbers of total lymphocytes, CD3^+^ cells and CD4^+^ T cells (Fig. 3C). When exposed to OAAH allergens, an approximately twofold increase in the total number of lymphocytes in the lungs of WT mice was observed (Fig. 3C). Among lymphocytes, the number of CD3^+^ T cells was clearly increased. This increase in T cells was not observed in OAAH-exposed JH^-/-^ mice. Furthermore, the number of CD4^+^ T cells was significantly lower in the lungs of OAAH-treated JH^-/-^ mice than in those of OAAH-treated WT mice (p<0.05). Altogether, these findings suggest that when mice are exposed repeatedly to multiple airborne allergens, the number of T cells and the amounts of Th2 cytokines in the lung tissues increase. These Th2-type immune responses appeared to be diminished in B cell-deficient mice.

### Allergen-driven development of Th2 cells is attenuated in B cell-deficient mice

**Fig 4:**
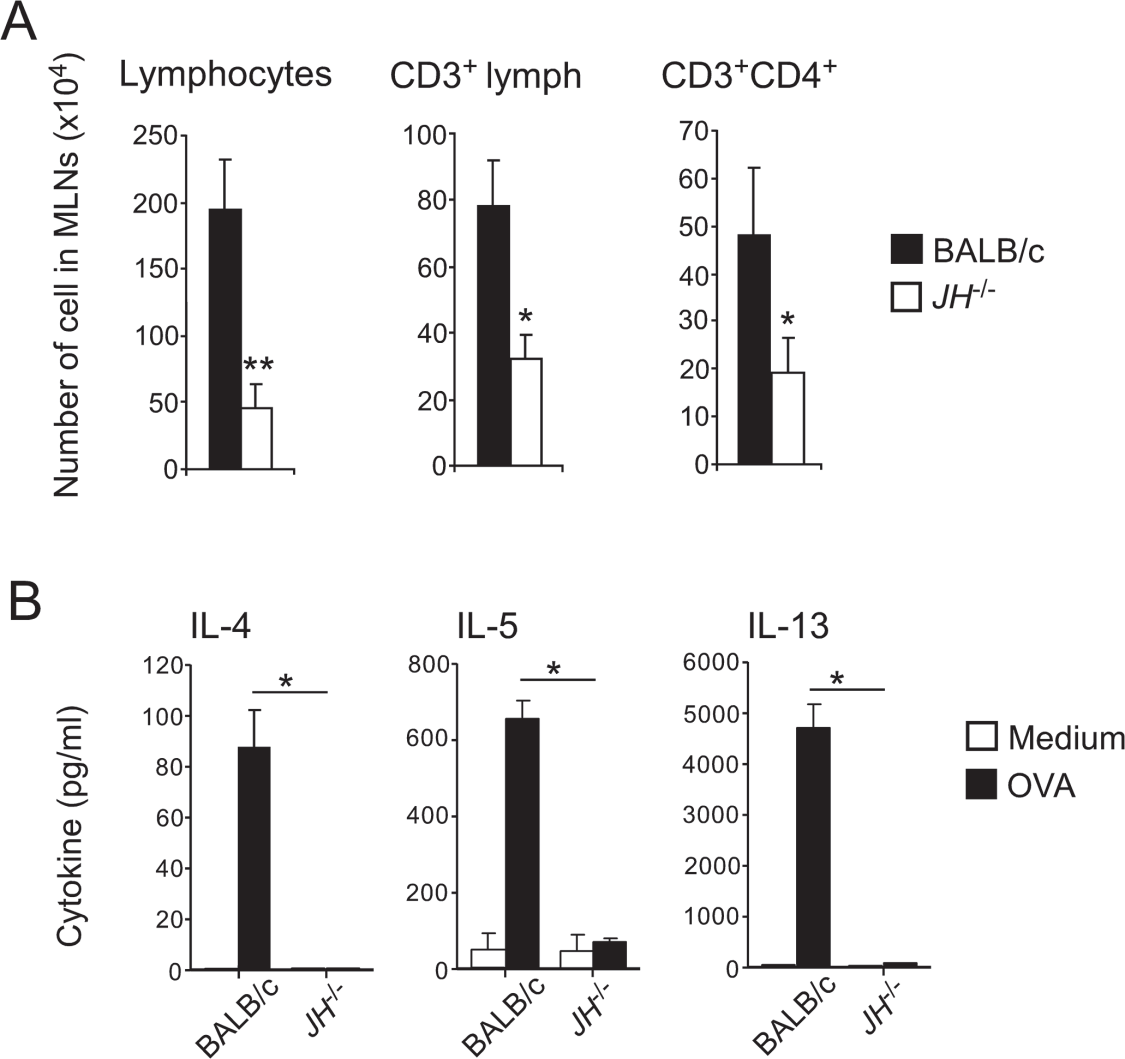
Th2-type responses in draining lymph nodes are attenuated in JH^-/-^ mice. WT or JH-^/-^ mice were intranasally exposed to OAAH for 2 weeks, as described in Fig. 1A. (A) Mediastinal lymph nodes (MLNs) were harvested, and cells were stained with anti-CD3 and anti-CD4 antibodies and analyzed by flow cytometry. Results are presented as mean±SEM (n = 5 mice per group). *, p<0.05; **, p<0.01. Data are representative of two independent experiments. (B) MLN cells were cultured with medium alone or with OVA (100 μg/ml) in 96-well tissue culture plates for 5 days. The levels of cytokines in the culture supernatants were analyzed by ELISA. *; p<0.05 between the groups indicated by horizontal lines. Data (mean±range, n = 2) are representative of three independent experiments.

Therefore, we examined whether B cells play any roles in the expansion and differentiation of antigen-specific T cells in lymphoid organs. Because the draining lymph nodes of the lungs, namely MLNs, were practically invisible in control mice that were exposed to PBS alone, we examined the MLNs that were obtained from OAAH-exposed mice. The numbers of total CD3^+^ lymphocytes and CD4^+^ T cells were significantly lower in MLNs from JH^-/-^ mice than in those from WT mice (Fig. 4A). When re-stimulated with OVA *in vitro*, MLN cells from OAAH-treated WT mice produced a large quantity of Th2 cytokines, including IL-4, IL-5, and IL-13, which suggests that T cells from these mice are sensitized to the OVA antigen that was included in the OAAH allergen cocktail. Indeed, no or minimal cytokines were detected when MLN cells were cultured without the antigen (i.e., medium). In contrast, MLN cells from OAAH-treated JH^-/-^ mice failed to produce detectable amounts of these Th2 cytokines after OVA re-stimulation *in vitro* (Fig. 4B). These findings suggest that B cells are required for the generation of optimal Th2-type immune responses in the lymphoid organs of animals that are exposed to common airborne allergens.

## Discussion

In this study, we found that B cells were required for airway inflammation, Th2 cytokine production, and AHR, which were induced by repeated exposures to common airborne allergens in mice. Although the contributions of B cells to the production of the IgE antibody in allergic diseases have been well established, the functional significance of B cells in the regulation of Th2-type immune responses has been controversial. For example, B cells are either not required, or are only partially required, for allergic airway inflammation or AHR in mice that were sensitized and challenged with a model antigen OVA or *Aspergillus* antigen [6,7,8,17,18,19]. In another study, B cell-deficient mice showed decreased airway levels of Th2 cytokines in response to cockroach allergens, but the magnitudes of airway inflammation were not affected [9]. A major difference in our animal model in this study, as compared to those published previously, is that we repeatedly exposed mice to a cocktail of multiple allergens, rather than a single allergen. This cocktail included HDM, *Alternaria*, and *Aspergillus*. In addition, mice were not sensitized previously by systemic immunization (e.g., intraperitoneal injection of the antigen). In this model, these allergens acted synergistically although *Alternaria* seems to provide the strongest contribution [10], and our current findings show that these allergens induced robust airway inflammation, pathological changes, and AHR in a B cell-dependent manner (Figs. 1 and 2).

In a mouse model using the model antigen OVA, neither IgE antibodies nor B cells are required for airway inflammation and AHR when mice are first immunized intraperitoneally, followed by airway challenge [17,18,19]. In contrast, B cells are required for AHR in mice that are only immunized via the airway [6], which suggests that the B-cell requirement may vary depending on the route of initial allergen exposure. The types of allergens may also produce differences. For example, when the *Aspergillus* extract is administered through the airways, similar levels of airway inflammation, AHR, and goblet cell hyperplasia are observed in B cell-deficient and WT mice [7,8]. However, exposure to the cockroach extract decreases AHR and Th2 cytokine production without affecting airway inflammation in B cell-deficient mice [9]. To mimic human allergen exposure in an experimental system, we exposed mice to allergens via the airway alone and used a cocktail of various allergens [10]. This unique model may have increased our ability to detect the functional importance of B cells. We speculate that a multiple-allergen and repeated-exposure model may require a variety of immune cell subtypes acting together to process and respond to complex allergens. In this type of setting, the need for B cells may become more apparent. Indeed, a recent study demonstrated that B cells can capture a cysteine protease papain, which is administered into the airways, and promote IL-4 expression by Th2 and follicular CD4^+^ T cells [20]. Protease activities from HDM and fungi are implicated in the development of Th2-type immune responses to these allergens [15]. Thus, B cells may play key roles in mediating Th2-type immune responses in response to protease allergens, especially when they are administered into the airways.

CD4^+^ T cells are generally essential for allergen-induced airway inflammation, and asthma is considered as a dysregulated Th2 response [3,4]. One of the novel observations in this study is that B cells are likely required for the differentiation and/or expansion of antigen-specific Th2-type CD4^+^ T cells in draining lymph nodes when the mice were exposed to multiple allergens (Fig. 4). The reduction in Th2 cytokine production and inflammation in the airways (Fig. 1), as well as the decrease in the number of CD4^+^ T cells in the lung tissues (Fig. 3), could be explained by the attenuated development of antigen-specific Th2 cells. The functional role of B cells in Th2 responses has been demonstrated in several model systems using helminth parasites and the experimental antigen, keyhole limpet hemocyanin [21,22,23,24,25]. In an experimental system using TCR transgenic mice, B cells present antigens and provide key co-stimulatory signals for T-cell expansion and differentiation toward the Th2-type response [23,24,26]. The observations from the current study add to this body of knowledge and suggest that B cells play critical roles in the optimal development of antigen-specific Th2 cells in response to exposures to common airborne allergens. However, we should also note that the culture of total MLN cells with OVA did not distinguish whether the attenuation in Th2 cytokine responses was due to either the impairment in Th2-cell development *in vivo* or an inadequate antigen presentation in the B cell-deficient culture *in vitro*.

How are B cells potentially involved in the development of Th2-type immune responses to inhaled allergens? T cells from B cell-deficient mice have been shown to produce levels of Th2 cytokines that are comparable to those from WT mice after OVA stimulation [6], suggesting that intrinsic defects of T cells are unlikely the case. In principle, B cells can modulate immune responses by producing antibodies, serving as antigen-presenting cells, providing co-stimulation signals, and secreting cytokines [27]. Therefore, the ability of B cells to drive antigen-induced Th2 responses can be explained by one or more of these mechanisms in various model systems [9,22,24,25,26,28,29]. For example, in a passive transfer model, hybridoma-derived IgE and IgG1 antibodies have been shown to play important roles in allergic airway eosinophilia and AHR [30]. More recently, dendritic cells produced IL-33, a critical cytokine for airway pathology induced by natural allergen exposure [10], when they were stimulated with immune complexes in vitro [31]. In our model system, OAAH exposure induced significant increases in total and OVA-specific IgE and IgG1 antibodies in WT mice but not in B cell-deficient JH^-/-^ mice (Fig. 3B). Therefore, it is possible that the absence of allergen-specific antibodies in JH^-/-^ mice contributed to the impaired development of Th2 responses in these mice. Future studies will be necessary to identify the specific immunological mechanism(s) by which B cells can initiate or enhance the development of allergen-specific Th2 cells in the airway mucosa when exposed to airborne allergens.

In summary, we found that B cells played critical role(s) in Th2-type immune responses, eosinophilic airway inflammation, and AHR when mice are exposed repeatedly to common airborne allergens. Because humans are constantly exposed to multiple airborne allergens [32,33], our results suggest that B cells are essential for the development of allergic immune responses in such settings. Our results also imply that pharmacological or immunological therapy that targets B cells may provide clinical benefits to prevent the development or exacerbation of allergic airway inflammation in patients with asthma.

## Supporting Information

S1 Fig Gating strategies for analyses of lymphocytes in lungs and MLNs. After exposure to OAAH allergens for 2 weeks, single-cell suspensions of the lungs and MLNs were stained with anti-CD3 and anti-CD4 antibodies and analyzed by flow cytometry. (A) Gating strategy for lung cells. (B) Gating strategy for MLN cells.
(TIF)
