# Supplementary figures and images for: B Cells Play Key Roles in Th2-Type Airway Immune Responses in Mice Exposed to Natural Airborne Allergens
Source: PLoS One. 2015 Mar 24;10(3):e0121660. doi: 10.1371/journal.pone.0121660 (PMC4372217; doi:10.1371/journal.pone.0121660)

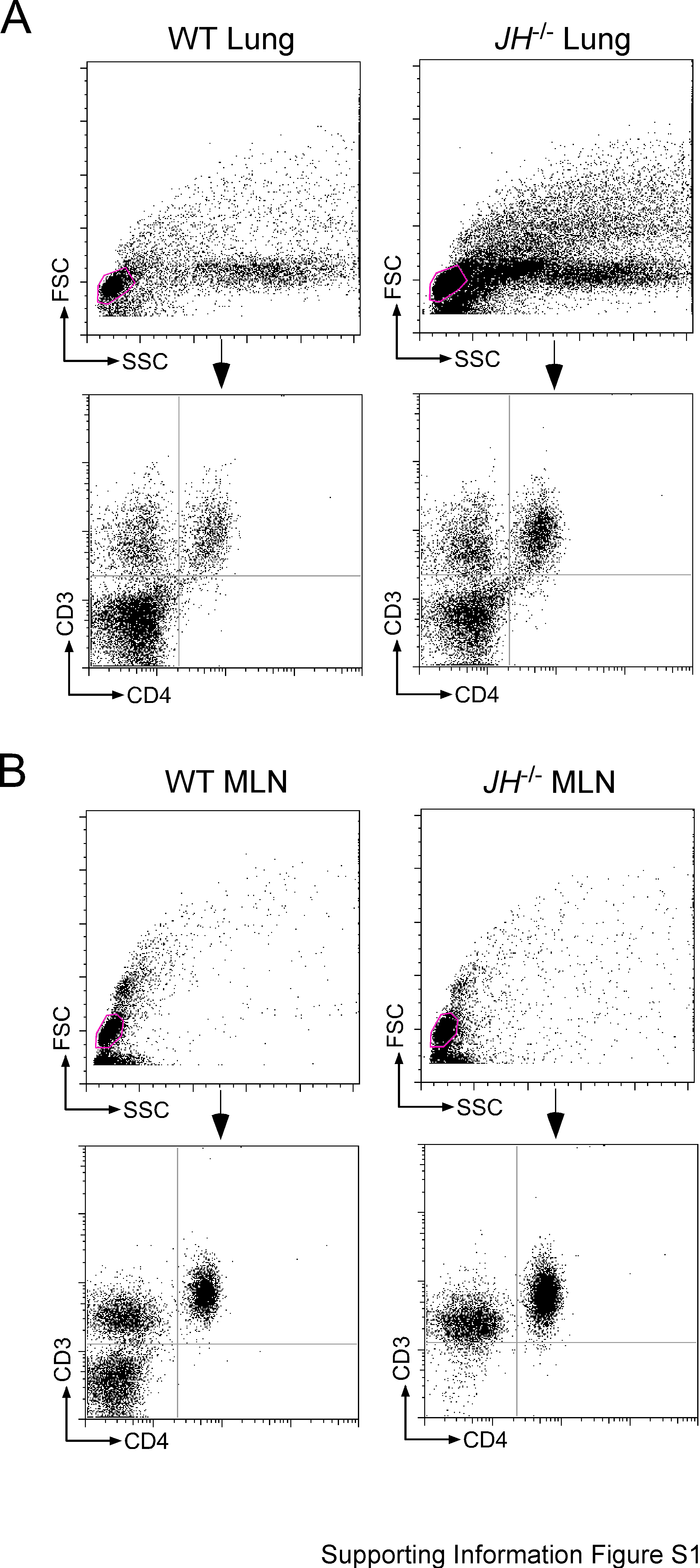

Supplement: S1 Fig — After exposure to OAAH allergens for 2 weeks, single-cell suspensions of the lungs and MLNs were stained with anti-CD3 and anti-CD4 antibodies and analyzed by flow cytometry. (A) Gating strategy for lung cells. (B) Gating strategy for MLN cells. (TIF) [file pone.0121660.s001.tif]
